# Supplementary material for: Biomolecular analyses reveal the age, sex and species identity of a near-intact Pleistocene bird carcass
Source: Commun Biol. 2020 Feb 21;3:84. doi: 10.1038/s42003-020-0806-7 (PMC7035339; doi:10.1038/s42003-020-0806-7)
Supplement: Supplementary file 1 — Description of Additional Supplementary Files [file 42003_2020_806_MOESM1_ESM.docx]

Supplementary Data 1

Concatenated cytb+ND2 sequences (2,034 bp)
