# Supplementary material for: Biomolecular analyses reveal the age, sex and species identity of a near-intact Pleistocene bird carcass
Source: Commun Biol. 2020 Feb 21;3:84. doi: 10.1038/s42003-020-0806-7 (PMC7035339; doi:10.1038/s42003-020-0806-7)
Supplement: Supplementary file 3 — Reporting Summary [file 42003_2020_806_MOESM3_ESM.pdf]

nature research

Corresponding author(s): Love Dalén

Last updated by author(s): 11.12.2019

Reporting Summary

Nature Research wishes to improve the reproducibility of the work that we publish. This form provides structure for consistency and transparency in reporting. For further information on Nature Research policies, see Authors & Referees and the Editorial Policy Checklist.

Statistics

For all statistical analyses, confirm that the following items are present in the figure legend, table legend, main text, or Methods section.

n/a	Confirmed	
☒	☐	The exact sample size (*n*) for each experimental group/condition, given as a discrete number and unit of measurement
☒	☐	A statement on whether measurements were taken from distinct samples or whether the same sample was measured repeatedly
☒	☐	The statistical test(s) used AND whether they are one- or two-sided<br>*Only common tests should be described solely by name; describe more complex techniques in the Methods section.*
☒	☐	A description of all covariates tested
☒	☐	A description of any assumptions or corrections, such as tests of normality and adjustment for multiple comparisons
☒	☐	A full description of the statistical parameters including central tendency (e.g. means) or other basic estimates (e.g. regression coefficient) AND variation (e.g. standard deviation) or associated estimates of uncertainty (e.g. confidence intervals)
☒	☐	For null hypothesis testing, the test statistic (e.g. *F*, *t*, *r*) with confidence intervals, effect sizes, degrees of freedom and *P* value noted<br>*Give P values as exact values whenever suitable.*
☒	☐	For Bayesian analysis, information on the choice of priors and Markov chain Monte Carlo settings
☒	☐	For hierarchical and complex designs, identification of the appropriate level for tests and full reporting of outcomes
☒	☐	Estimates of effect sizes (e.g. Cohen's *d*, Pearson's *r*), indicating how they were calculated

Our web collection on statistics for biologists contains articles on many of the points above.

Software and code

Policy information about availability of computer code

Data collection

no code was use for data collection

Data analysis

Oxcal computer program (v4.3)(Ramsey & Lee, 2013)
bcl2Fastq v2.17.1
SeqPrep v1.1(John JS, 2011)
BWA 0.7. (Li & Durbin, 2010)
SAMtools v1.9 (Li, 2011)
Picard v1.141 (http://broadinstitute.github.io/picard)
GATK v3.4.0 (McKenna et al., 2010)
Geneious® v7.0.336 (Kearse et al., 2012)
BLASTn (Altschul, Gish, Miller, Myers, & Lipman, 1990; Johnson et al., 2008)
Mitobim v1.9.0 (Hahn, Bachmann, & Chevreux, 2013)
BEAST2 v2.4.0 (Bouckaert et al., 2014)
Tracer v.1.6 (Rambaut, Suchard, Xie, & Drummond, 2014)
TreeAnnotator v.1.7.5 (Rambaut & Drummond, 2013)
FigTree v.1.4.3 (Rambaut, 2007)

References

Altschul, S. F., Gish, W. R., Miller, W., Myers, E. W., & Lipman, D. J. (1990). Basic local alignment search tool. Journal of Molecular Biology, 215(3), 403–410. Retrieved from http://linkinghub.elsevier.com/retrieve/pii/S0022283605803602
Bouckaert, R., Heled, J., Kühnert, D., Vaughan, T., Wu, C. H., Xie, D., … Drummond, A. J. (2014). BEAST 2: A Software Platform for Bayesian Evolutionary Analysis. PLoS Computational Biology, 10(4). doi:10.1371/journal.pcbi.1003537
Hahn, C., Bachmann, L., & Chevreux, B. (2013). Reconstructing mitochondrial genomes directly from genomic next-generation

sequencing reads - A baiting and iterative mapping approach. Nucleic Acids Research, 41(13). doi:10.1093/nar/gkt371
John JS. (2011). SeqPrep 1.1. Https://Github.Com/Jstjohn/ SeqPrep.
Johnson, M., Zaretskaya, I., Raytselis, Y., Merezhuk, Y., McGinnis, S., & Madden, T. L. (2008). NCBI BLAST: a better web interface. Nucleic Acids Research, 36(Web Server issue). doi:10.1093/nar/gkn201
Kearse, M., Moir, R., Wilson, A., Stones-Havas, S., Cheung, M., Sturrock, S., … Drummond, A. (2012). Geneious Basic: An integrated and extendable desktop software platform for the organization and analysis of sequence data. Bioinformatics, 28(12), 1647–1649. doi:10.1093/bioinformatics/bts199
Li, H. (2011). A statistical framework for SNP calling, mutation discovery, association mapping and population genetical parameter estimation from sequencing data. Bioinformatics, 27(21), 2987–2993. doi:10.1093/bioinformatics/btr509
Li, H., & Durbin, R. (2010). Fast and accurate long-read alignment with Burrows-Wheeler transform. Bioinformatics, 26(5), 589–595. doi:10.1093/bioinformatics/btp698
McKenna, A., Hanna, M., Banks, E., Sivachenko, A., Cibulskis, K., Kernytsky, A., … DePristo, M. A. (2010). The Genome Analysis Toolkit: A MapReduce framework for analyzing next-generation DNA sequencing data. Genome Research, 20, 1297–1303. doi:10.1101/gr.107524.110.20
Rambaut, A. (2007). FigTree, a graphical viewer of phylogenetic trees. See Http://Tree.Bio.Ed.Ac.Uk/Software/Figtree.
Rambaut, A., & Drummond, A. (2013). TreeAnnotator v1.7.0. University of Edinburgh, Institute of Evolutionary Biology.
Rambaut, A., Suchard, M., Xie, D., & Drummond, A. (2014). 'Tracer' 1.6. Available at: http://beast.bio.ed.ac.uk/Tracer.
Ramsey, C. B., & Lee, S. (2013). Recent and Planned Developments of the Program OxCal. Radiocarbon, 55(2), 720–730. doi:10.1017/s0033822200057878

For manuscripts utilizing custom algorithms or software that are central to the research but not yet described in published literature, software must be made available to editors/reviewers. We strongly encourage code deposition in a community repository (e.g. GitHub). See the Nature Research guidelines for submitting code & software for further information.

Data

Policy information about availability of data

All manuscripts must include a data availability statement. This statement should provide the following information, where applicable:

- Accession codes, unique identifiers, or web links for publicly available datasets
- A list of figures that have associated raw data
- A description of any restrictions on data availability

all raw genomic data will be publicly available on ENA (study number: PRJEB35255)
Figures:
Figure 1. (a, b) Dorsal and ventral view of the c. 46 ka old bird carcass recovered from the Siberian permafrost. (c) Map indicating its sampling location (red dot).
Figure 2. Bayesian phylogeny for the concatenated ND2 and cytb (2034 bp) for 31 horned lark from Ghorbani et al.20 and the ancient horned lark highlighted in grey. Nodes with <0.5 support should be considered collapsed.

Field-specific reporting

Please select the one below that is the best fit for your research. If you are not sure, read the appropriate sections before making your selection.

☐ Life sciences ☐ Behavioural & social sciences ☒ Ecological, evolutionary & environmental sciences

For a reference copy of the document with all sections, see nature.com/documents/nr-reporting-summary-flat.pdf

Ecological, evolutionary & environmental sciences study design

All studies must disclose on these points even when the disclosure is negative.

Study description	This study uses ancient DNA techniques to identify the age, sex and species identity of a near-intact Pleistocene bird carcass
Research sample	The feather and tissue sample used was taken from near-complete bird carcass found in Siberia
Sampling strategy	The sample was obtained opportunistically from a permafrost tunnel
Data collection	Boris Berezhnov and Spartak Khabrov found the bird remains
Timing and spatial scale	only one sample alone dating back some 46 ka BP was obtained
Data exclusions	NA
Reproducibility	NA
Randomization	NA
Blinding	NA

Did the study involve field work? ☐ Yes ☒ No

Reporting for specific materials, systems and methods

We require information from authors about some types of materials, experimental systems and methods used in many studies. Here, indicate whether each material, system or method listed is relevant to your study. If you are not sure if a list item applies to your research, read the appropriate section before selecting a response.

Materials & experimental systems

n/a	Involved in the study
☒	☐ Antibodies
☒	☐ Eukaryotic cell lines
☐	☒ Palaeontology
☐	☒ Animals and other organisms
☒	☐ Human research participants
☒	☐ Clinical data

Methods

n/a	Involved in the study
☒	☐ ChIP-seq
☒	☐ Flow cytometry
☒	☐ MRI-based neuroimaging

Palaeontology

Specimen provenance	Nort-estearn SIberia, Tirekhtyakh River, Belaya Gora
Specimen deposition	Sakha Academy of Sciences in Yakutsk (accession ID: 2018-Tir-Shorelark-01)
Dating methods	radiocarbon dating was performed at the Oxford Radiocarbon Accelerator Unit (ORAU) using the Oxcal computer program (v4.3)

☒ Tick this box to confirm that the raw and calibrated dates are available in the paper or in Supplementary Information.

Animals and other organisms

Policy information about studies involving animals; ARRIVE guidelines recommended for reporting animal research

Laboratory animals	NA
Wild animals	Near-intact bird carcass found in permafrost
Field-collected samples	NA
Ethics oversight	NA

Note that full information on the approval of the study protocol must also be provided in the manuscript.
